# Role of Secreted Frizzled-Related Protein 1 in Early Mammary Gland Tumorigenesis and Its Regulation in Breast Microenvironment

**DOI:** 10.3390/cells9010208

**Published:** 2020-01-14

**Authors:** Alisson Clemenceau, Caroline Diorio, Francine Durocher

**Affiliations:** 1 Department of Molecular Medicine, Faculty of Medicine, Laval University, Quebec, QC G1T 1C2, Canada; alisson.clemenceau.1@ulaval.ca; 2 Cancer Research Centre, CHU de Quebec Research Centre, Quebec, QC G1V 4G2, Canada; Caroline.Diorio@crchudequebec.ulaval.ca; 3 Department of Preventive and Social Medicine, Faculty of Medicine, Laval University, Quebec, QC G1T 1C2, Canada

**Keywords:** breast cancer, *secreted frizzled-related protein 1*, *SFRP1*, lobular involution, breast involution, microcalcifications, inflammation, apoptosis, osteomimic microenvironment, osteoblast-like cells

## Abstract

In mice, the lack of *secreted frizzled-related protein 1* (*SFRP1*) is responsible for mammogenesis and hyperplasia, while, in bovines, its overexpression is associated with post-lactational mammary gland involution. Interestingly, there are no reports dealing with the role of *SFRP1* in female involution. However, *SFRP1* dysregulation is largely associated with human tumorigenesis in the literature. Indeed, the lack of *SFRP1* is associated with both tumor development and patient prognosis. Considering the increased risk of breast tumor development associated with incomplete mammary gland involution, it is crucial to demystify the “grey zone” between physiological age-related involution and tumorigenesis. In this review, we explore the functions of *SFRP1* involved in the breast involution processes to understand the perturbations driven by the disappearance of *SFRP1* in mammary tissue. Moreover, we question the presence of recurrent microcalcifications identified by mammography. In bone metastases from prostate primary tumor, overexpression of *SFRP1* results in an osteolytic response of the tumor cells. Hence, we explore the hypothesis of an osteoblastic differentiation of mammary cells induced by the lack of *SFRP1* during lobular involution, resulting in a new accumulation of hydroxyapatite crystals in the breast tissue.

## 1. Introduction

**Figure 1:**
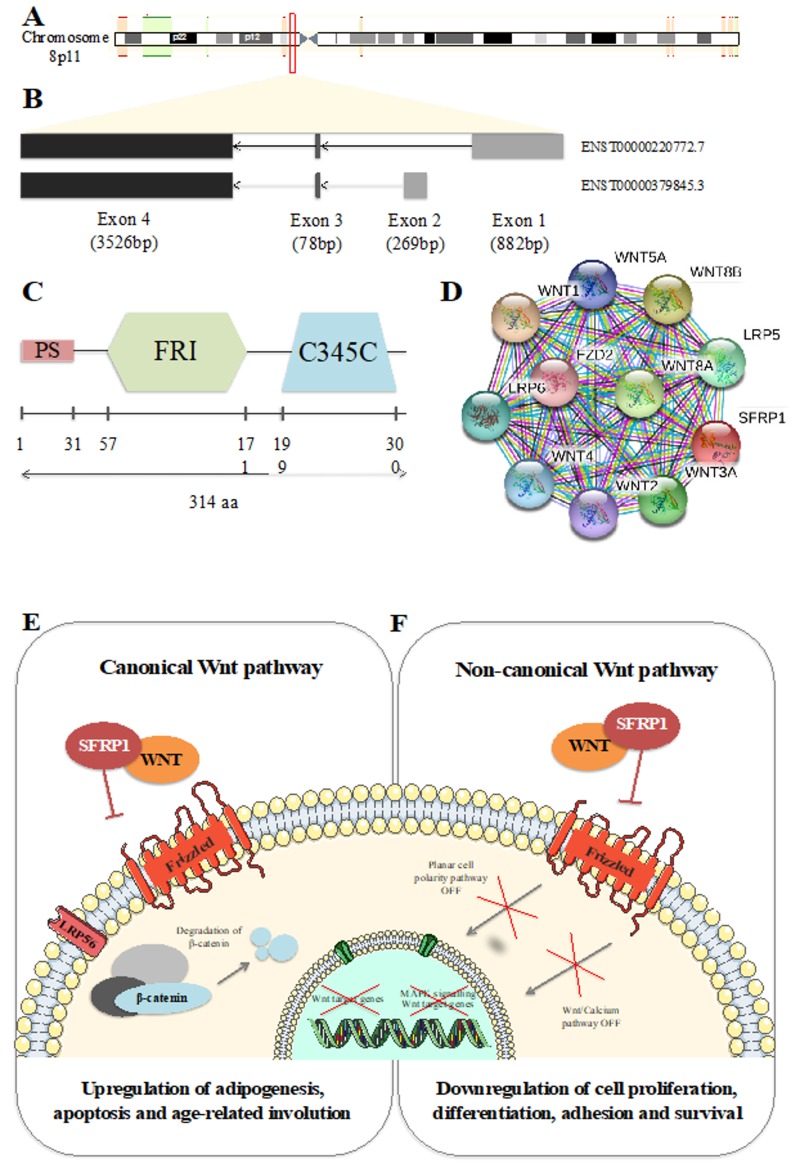
*Secreted frizzled-related protein 1* (*SFRP1*) identity. (**A**) Schematic representation of *SFRP1* position on chromosome 8. (**B**) Schematic representation of *SFRP1* isoforms. (**C**) Schematic representation of SFRP1 protein domains. (**D**) Top 10 of protein–protein interactions between SFRP1 and Wnt signaling pathways. Figure drawn using Simple modular architecture research tool (SMART) [7]. Wnt canonical (**E**) and non-canonical (**F**) pathways in physiological context.

*Secreted frizzled-related protein 1* (*SFRP1*) encodes one of the eight known SFRP family members. This 3873 base pair coding gene is localized on chromosome 8p11, from nucleotide 41,261,957 to 41,309,473 (Figure 1A). Composed of 3 exons, it can be spliced into two different isoforms described in Figure 1B. Translation of *SFRP1* results in a 314 amino acid (aa) protein composed of three major domains, schematized in Figure 1C. The first domain (1 to 31 aa) is a peptide signal (PS) that allows the protein to be transported to the extracellular matrix after synthesis. The second domain (57 to 171aa), the frizzled (FRI), is an extracellular cysteine rich domain (CRD) largely conserved among species [1]. It is composed of 10 cysteines forming disulfide bonds visible on the alpha-helical crystal structure of the domain [2]. Due to the fact of its homology, the *SFRP1* CRD binds the Wnt-binding site of frizzled proteins (Fz) [3,4,5]. Bafico et al. [6] reported that *SFRP1* is also able to directly interact with Fz resulting in a non-functional complex.

## 2. *SFRP1* in Wnt Signaling Pathway

In 1997, Finch et al. [8] described *SFRP1* as a secreted Wnt antagonist. In fact, *SFRP1* PS allows the protein to be transported to the extracellular matrix. In this compartment, *SFRP1* fixes Wnt molecules, inducing a downregulation of the Wnt signaling pathway. The Wnt molecules caption induces a decrease of the intracellular β-catenin level which is responsible for the downregulation of Wnt target genes’ transcription. As a primordial regulator of cell growth, cell polarity, cell fate determination, and malignant transformation, *SFRP1* is widely expressed in human cells [8]. Later, in 2000, Uren et al. [9] reported that low concentration of SFRP1 could potentiate Wnt signaling pathway activity rather than inhibiting it. This result suggests the existence of a high-affinity site of liaison involved in low SFRP1 concentration and conversely a low affinity site of liaison in high SFRP1 concentration. *Secreted frizzled-related protein 1* (*SFRP1*) is known to downregulate canonical Wnt pathway. Moreover, the important homology between Wnt molecules suggests that *SFRP1* could be able to regulate the Wnt non-canonical pathway as well (Figure 1D–F). In fact, in prostate epithelial cells, the overexpression of *SFRP1* had no impact on intracellular β-catenin level. However, it activated the WNT/JNK pathway [10]. To date, the processes used by *SFRP1* to regulate cell activity remain poorly understood. Nevertheless, the Wnt signaling pathway, highly conserved during evolution, has a crucial role in embryonic development and in adult tissues proliferation, differentiation, and apoptosis. Because of these crucial roles, dysregulation of the Wnt signaling pathway antagonists is strongly associated with many diseases and most importantly cancer [11,12,13].

## 3. *SFRP1* Expression Is Deregulated in Breast Cancer

Many evidence that *SFRP1* dysregulation is involved in tumorigenesis is available in the literature [10,14,15,16,17,18,19,20,21,22,23,24,25,26,27,28,29,30,31,32,33,34,35,36,37]. Hence, understanding the mechanism of *SFRP1* remains essential in order to develop cancer treatments. Several studies have demonstrated that *SFRP1* in breast cancer is downregulated at both the mRNA and protein levels (Table 1). Moreover, this downregulation is also associated with poor survival outcome [25]. It has also been reported that *SFRP1* is wildly downregulated in breast malignant lesions. Under this scenario, the activation of canonical and non-canonical Wnt pathways depends on breast cancer subtypes or vice versa [29]. In fact, Huth et al. [29] reported that the canonical Wnt pathway is affected in basal-like breast cancer while the non-canonical Wnt pathway is affected in luminal-like breast cancer cells. This diversity in the operating mode makes the underlying molecular mechanisms of *SFRP1* particularly difficult to understand. However, the studies described in Table 1 reported that *SFRP1* is under-expressed in breast cancer tissue compared to normal tissue. In fact, as a down-regulator of the Wnt signaling pathway, *SFRP1* is involved in the negative control of cell proliferation, differentiation, and survival. In other words, *SFRP1* is a tumor-suppressor in physiological conditions. Furthermore, in triple-negative breast cancer cases, the over-expression of *SFRP1* is responsible for an increasing sensitivity to chemotherapy [32]. By its pro-apoptotic effect, *SFRP1* is a potential therapeutic tool that should be further investigated. Interestingly, if we focus specifically on breast tissue, the downregulation of *SFRP1* in both human and mice *SFRP1* ^−/−^ is responsible for the increasing estrogen-induced response and hyperplasia [30]. In normal mammary epithelial cells, estrogen positively regulates *SFRP1* expression [25]. A genome-wide association study reported that, in the *SFRP1*-modulated gene interaction network, 27 genes were modulated in a cluster involved in estrogen stimulus response [31]. To exacerbate the evidence of estrogen pathway involvement in *SFRP1*-related breast tumorigenesis, Bernemann et al. [32] showed that *SFRP1* is overexpressed in triple-negative breast tumors compared with other breast tumors. The relationship between *SFRP1* and estrogen is an important facet to investigate. Indeed, breast evolution during a woman’s life is hormone-dependent. Furthermore, estrogen decreases dramatically during menopause which is responsible for a negative regulation of the bone resorption and formation, resulting in loss of bone mass [33,34]. That decrease of bone mass was associated with the increasing expression of miR-542-3p, involved in the downregulation of osteoblast differentiation by targeting *SFRP1* in rats after ovariectomy-related osteoporosis [35]. Understanding the cross-regulation between *SFRP1* and estrogen sensitivity could allow us to better understand both breast tumorigenesis and menopause-related osteoporosis.

### Hypermethylation of SFRP1

To better understand which process induces *SFRP1* expression dysregulation, some studies focused on the epigenetic profile of the gene. As described in Table 1, *SFRP1* downregulation is associated with the hypermethylation of the *SFRP1* promoter region’s CpG islands, suggesting that epigenetics could be as crucial as gene aberrations in breast tumorigenesis [15,26,27,28]. In 2006, Veeck et al. [28] reported that the hypermethylation of *SFRP1* promoter was the principal cause of gene silencing in breast cancer. Furthermore, they associated this hypermethylation to an unfavorable prognosis for patients. This was also demonstrated in 2007 in renal cell carcinomas by Dahl et al. [15] suggesting that the hypermethylation of *SFRP1* promoter contributes to the development of multiple human tumors. Later in 2012, Mukherjee et al. [27] reported that 67% of breast cancer samples were altered in *SFRP1*. In the same cohort, 56% of samples were hypermethylated in the *SFRP1* promoter region, suggesting once more that the hypermethylation of *SFRP1* promoter is responsible for *SFRP1* silencing in breast cancer [27]. More recently, the integration of expression data and next-generation sequencing allowed Li et al. [26] to characterize the correlation between *SFRP1* promoter methylation and gene expression regulation. They demonstrated a strong negative correlation between both, suggesting that hypermethylation of *SFRP1* promoter is responsible for *SFRP1* silencing in breast cancer. Furthermore, they reported that this methylation quantification was a better parameter to improve the diagnosis of the disease [26]. The *SFRP1* epigenetic seems to be a crucial player in early breast tumorigenesis and needs to be explored to better understand the mechanisms of tumor development. 

## 4. *SFRP1* Has a Major Role in the Lobular Involution Process

### 4.1. Involution

**Figure 2:**
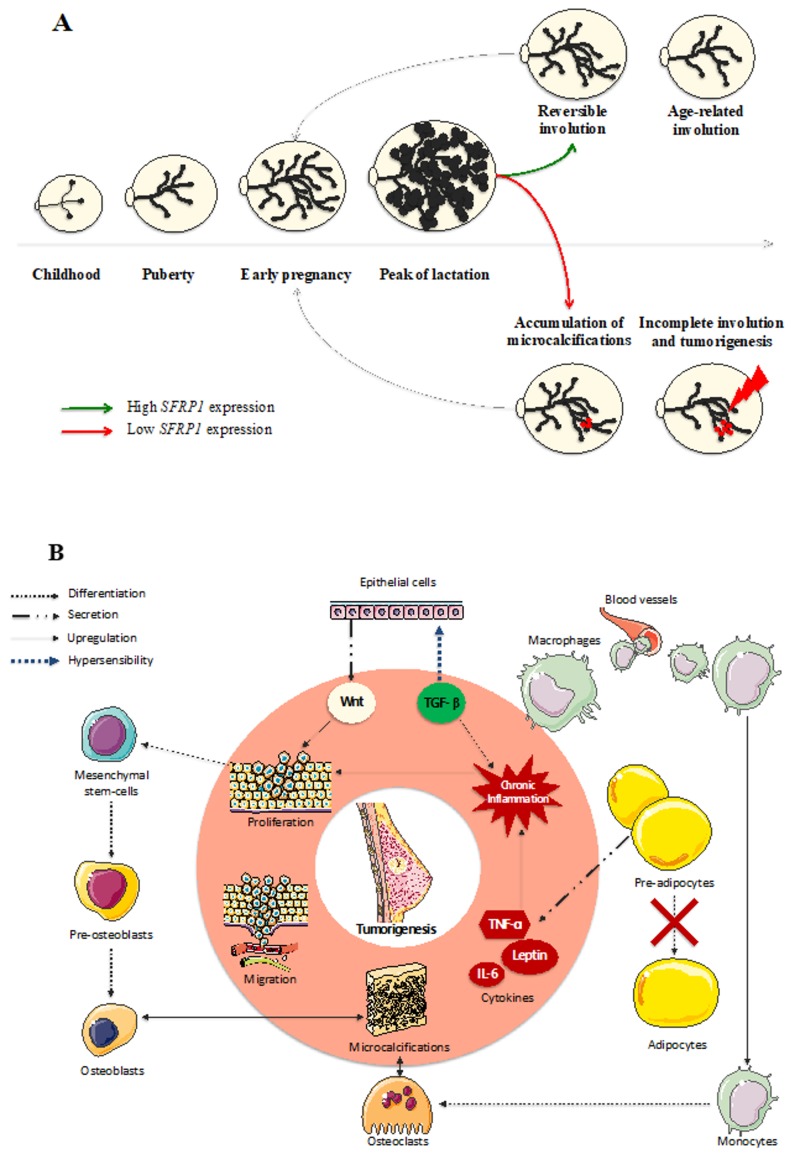
*SFRP1* downregulation in breast tissue induces a chain reaction responsible for the malignant transformation of the cells. (**A**) Mammary glands evolve throughout a woman’s life until reaching their growth peak during breastfeeding. Afterwards, acinis remain useful, so the breast starts a reversible involution to destroy the excessive glandular and epithelial tissue. During the peri-menopausal step of a woman’s life, another involution appears to eliminate the rest of the useful tissue, decreasing at the same time the risk of breast cancer development. As a down-regulator of cell growth, *SFRP1* under-expression could be responsible for the incomplete age-related breast involution resulting in a malignant transformation of the breast in the presence of microcalcifications. (**B**) The over-activation of the Wnt signaling pathway due to the *SFRP1* down-regulation in breast tissue is responsible for the increasing proliferation and migration of mammary cells. The local inflammation needed to start the physiological age-related breast involution is responsible for the recruitment of the immune system. In the presence of microcalcifications, monocytes are able to differentiate in osteoclasts, while mesenchymal stem cells can differentiate in osteoblasts responsible for de novo microcalcifications formation. Furthermore, the replacement of epithelial tissue by adipose tissue needs pre-adipocytes maturation which is downregulated by the under-expression of *SFRP1*. The production of adipokines by immature adipocytes generates a chronic inflammation exacerbated by the hypersensitivity of epithelial cells to TGF- β due to the *SFRP1* under-expression.

Mammary glands are continuously evolving during a woman’s life and hormone cycles (Figure 2A). In the course of lobular evolution steps, lobular involution in both post-lactation and peri-menopause contexts is strongly associated with breast cancer risk. Indeed, many studies have demonstrated an inverse relationship between the degree of lobular involution and breast cancer risk in both normal and benign breast lesions [38,39,40,41,42]. The word “involution” is commonly used to describe an inward folding. In the breast tissue, the term “involution” can be used to describe the regression of the mammary gland. Such process occurs at the end of the breastfeeding period, to return to the pre-pregnancy mammary gland size. During this reversible involution, useless acinis are degraded by the immune system and replaced by collagen and adipose tissue. During the peri-menopausal period, an irreversible involution of the mammary gland occurs. In fact, with the impossibility to procreate comes the futility of milk production by the breast. To limit the risk of malignant transformation, useless epithelial cells are eliminated from the breast by the immune system and replaced by adipose tissue. This process remains poorly understood, notably because of the important intrapersonal variability. In fact, some women benefit from a total disappearance of types 2–3 lobules, resulting in a total absence of acinis at the end of the lobular involution, while others have an incomplete mammary gland regression increasing their number of epithelial cells in the breast tissue hence increasing, at the same time, their risk in developing a breast tumor. There is as yet no study reporting evidence of a relationship between *SFRP1* and lobular involution in women. However, *SFRP1* appears to have a major impact on breast hyperplasia in mice, as Gauger et al. [43] reported that, in virgin knock-down *SFRP1* ^−/−^ mice, the ductal branching and the lobulo-alveolar activity of the breast was comparable to mid-pregnant wild-type mice. On the other hand, Zheng et al. [44] described that, in bovines, *SFRP1* is overexpressed in the late lactation process compared to the early peak of lactation, suggesting the implication of *SFRP1* in reversible lobular involution following lactation. Taken together, these results suggest a causative role of *SFRP1* in both early regulation of breast development and reversible post-lactation involution. Regarding a potential role of *SFRP1* in breast involution and considering the protective role of mammary involution against breast tumorigenesis [39,40,41,42,45,46,47,48,49,50,51], *SFRP1* could be a crucial player, to optimize lobular involution and decrease breast cancer risk. To better understand the role of *SFRP1* in breast involution, we describe the implication of this protein in each molecular step involved in the lobular involution hereafter.

### 4.2. Adipogenesis

The mammary gland involution is described by the gradual replacement of breast epithelium and stroma by collagen and adipose tissue and by the regression of type 2–3 lobules to type 1 lobules [52]. Many studies focus on obesity-related unbalance resulting in the disturbed secretion of inflammatory molecules named adipokines by the adipose tissue [53,54,55,56,57,58]. Interestingly, adipogenesis also occurs during breast involution, so that adipokines overexpression could appear during this specific step. In fact, the interaction between cancer cells and adipocytes could result in their reprogramming in cancer-associated adipocytes (CAAs), responsible for an abundant secretion of adipokines which upregulate the adhesion, migration, and invasion of the cells [54,55]. Furthermore, in mice, evidence of adipocytes delipidation to produce de novo lipids by lactating epithelial cells was reported [54,59]. Co-cultures of adipocyte stem cells with MCF7 cells showed an enhancing proliferation of the tumoral cells due to the estrogen-activated response mediated through leptin [53]. Interestingly, in mice adipocytes, *SFRP1* expression increases during adipogenesis [60]. In humans, *SFRP1* is also upregulated in early adipogenesis [61], both at the mRNA and protein levels. Legathu et al. [61] reported that *SFRP1* expression is increased dramatically following induction of adipocytes differentiation. This *SFRP1* overexpression induces the decrease of intracellular β-catenin levels, suggesting that adipogenesis is activated by the negative regulation of the Wnt canonical signaling pathway by *SFRP1* [61]. In *SFRP1* ^−/−^ mice, when obesity is induced, there is an increase in the inflammatory response associated with adipogenesis, and an upregulation of macrophage activity [62]. This inflammatory exacerbation is also associated with mammary branching [63]. Furthermore, body weight gain in post-menopausal women is associated with higher breast cancer risk [64,65]. Age-related weight gain in women, combined with the replacement of breast epithelium by adipose tissue during involution suggest that adipose tissue could play an important role in early tumorigenesis. In the case of an abnormal under-expression of *SFRP1*, we can hypothesize that pre-adipocytes and adipocytes stem cells are not able to differentiate in mature adipocytes, resulting in an overexpression of cytokines and adipokines responsible for a chronic inflammation of the breast tissue. The incomplete involution potentially associated with the lack of *SFRP1* results in the presence of numerous epithelial cells able to delipidate adipocytes to make more energy. That lack of *SFRP1* results also in the hyper-activation of the Wnt signaling pathway, which is responsible for enhancing cell proliferation, adhesion, and survival. The *SFRP1* gene seems crucial at many steps for maintaining the balance between normal adipose tissue remodeling and tumorigenesis.

### 4.3. Inflammation

The *SFRP1* gene is upregulated in physiological inflammatory conditions, such as during post-lactation involution, suggesting a role in the degradation of acini by the immune system [66]. Interestingly, in periodontal tissue, the inhibition of SFRP1 at the protein level with a specific antibody is associated with less inflammation and a significant reduction of apoptosis in periodontitis context [67]. The existence of a crosslink between Wnt signaling pathway and TGF-β signaling pathway seems to be the explanation of such cross-regulations between inflammation and the Wnt signaling pathway. In fact, the under-expression of *SFRP1* in non-malignant cell lines results in the increasing sensitivity of the cell to the anti-inflammatory molecule TGF-β [68]. Furthermore, Dzialo et al. [69] proposed a model in which TGF-β activates the Wnt canonical signaling pathway by producing Wnt molecules. Interestingly, chronic inflammation of the breast tissue is inversely associated with lobular involution [52]. This suggests that anti-inflammatory processes could also promote cell proliferation and differentiation. In a breast cancer context, the accumulation of Wnt molecules due to the TGF-β pathway, combined with the lack of *SFRP1*, result in a dramatic over-activation of the Wnt signaling pathway and, consequently, in an increasing cell proliferation (Figure 2B). This suggests a sensitive balance between the inflammation needed to initiate the destruction of the epithelial cells by the immune system and the chronic inflammation responsible for decreasing breast involution and increasing hyperplasia. We hypothesize that the lack of *SFRP1* is responsible for an upregulation of TGF-β sensitivity which is responsible for the increase of Wnt molecule production and so—an abnormal hyperplasia. The combination of increasing TGF-β sensitivity and adipocyte-related inflammation could result in chronic inflammation resulting in an incomplete breast involution and a higher risk of breast tumorigenesis. Furthermore, high estrogen levels decrease immune reaction in the breast tissue [70]. This evidence suggests that a post-menopausal lobular involution could result in a higher risk of chronic inflammation, promoting breast tumorigenesis.

### 4.4. Apoptosis

Lobular involution is also characterized by both apoptosis of epithelial cells and lobulo-alveolar remodeling [71,72]. Regarding apoptosis, few studies have reported an association between *SFRP1* expression and apoptosis in breast tissue. In fact, the lack of *SFRP1* affects apoptotic gene expression and activity, suggesting a major role for this gene in the regulation of cell survival and proliferation in the breast [73]. In human colorectal cancer cell lines, Wang et al. [23] demonstrated that *SFRP1* overexpression promotes apoptosis. Similar results have been reported in a cholangiocarcinoma context using miR-191 to knockdown *SFRP1* [74]. In bone marrow-derived mesenchymal stem cells, the use of miR-144 targeting *SFRP1* inhibited apoptosis as well [75]. Genome-wide identification of key modulators implicated in breast tumorigenesis demonstrated a significant effect of immunity processes in breast tumor development. In fact, among the top clusters of gene ontology (GO) [76,77] terms enriched in modulator genes, the majority were directly involved in immune cell activation, apoptosis, and inflammatory response to immunity [29,31,78]. Furthermore, another cluster highlighted by these studies is involved in tissue remodeling, specifically in cell adhesion. Moreover, Chiu et al. [31] focused their analyses on specific *SFRP1*-modulated genes in breast cancer, and among the top six clusters of GO [76,77] terms, four were involved in tissue remodeling like extracellular structure or cytoskeleton organization. Regarding the top cluster enriched for microRNAs in human primary breast nodules, Yang et al. [78] concluded, once more, that apoptosis and inflammation were dramatically dysregulated. These results taken together suggest that the lack of *SFRP1* in breast could be responsible for a downregulation of epithelial cells apoptosis and tissue remodeling which is responsible for an incomplete involution associated with an increasing breast cancer risk and, in worst cases, in an hyperplasia responsible for the early development of breast tumor.

## 5. *SFRP1* Expression Dysregulation Is Responsible for an Osteoblastic Differentiation of Breast Cells and an Accumulation of Microcalcifications

### 5.1. SFRP1 and Osteoblast-Like Cells in the Breast

The *SFRP1* gene is a well-known important regulator of bone remodeling. Indeed, *SFRP1* is expressed by osteoblasts and inhibits osteoclast formation through its binding with receptor activator of nuclear factor kappa-B ligand (RANKL) [79,80]. In human fetal osteoblastic cell lines, the inhibition of *SFRP1* resulted in the promotion of osteoblastic differentiation due to the activation of the Wnt/β-catenin signaling pathway [81]. Tang et al. [75] demonstrated in bone marrow-derived mesenchymal stem cells that a decrease in *SFRP1* expression using miR-144 induces osteoblastic differentiation of the cells. Interestingly, the lack of *SFRP1* also seems to be involved in cells activity. Once differentiated, the lack of *SFRP1* is responsible for the upregulation of trabecular bone formation by the osteoblasts in mice [82]. Furthermore, many microRNAs-related dysregulations in breast tumor concern the bone morphogenic protein response, vitamin D response, and osteoblasts proliferation [78]. These pathways are directly related to the phospho-calcium homeostasis [83,84,85]. Furthermore, vitamin D is also known as a down-regulator of the Wnt pathway and an up-regulator of the TGF-β pathway, suggesting a crosstalk between bone homeostasis, inflammation, and apoptosis [86] that could be involved in breast cancer tumorigenesis. Kothari et al. [37] described another evidence of this potential osteoblast differentiation in the breast. The authors performed transcriptome analyses of breast lesions at different stages of tumor aggressiveness. Expression of *SFRP1* decreases with tumor progression. Interestingly, this decrease is accompanied with the increase of *Secreted Phosphoprotein 1* (*SPP1*) and *Periostin* (*POSTN*) expression [37]. *Secreted Phosphoprotein 1*, also named *Osteopontin*, is expressed by osteoblasts to promote bone resorption by increasing osteoclasts bone adhesion [87,88]. *Periostin* is also produced by osteoblasts and plays a preponderant role in tissue development and regeneration including wound healing [89,90]. In the context of inflammatory bone disease, resolving the resorption-induced inflammation by decreasing *SFRP1* expression induces the Wnt canonical signaling pathway activation which is essential for osteoblasts differentiation and activity [91,92,93,94]. With this evidence, we hypothesize that osteoblast differentiation in the breast tissue could result in a de novo bone construction.

### 5.2. SFRP1 and Microcalcifications

Breast microcalcifications are present in 30% to 50% of all malignant breast lesions. Most breast calcifications visible at mammography are made of a combination of calcium and phosphate, the exact composition of the mineral part of bones. Moreover, evidences of osteoblast-like cells in breast tissue suggest a potential for breast cells differentiation in a microcalcification environment [37,95,96,97,98,99,100,101,102,103]. In 2005, Morgan et al. [99] reported that microcalcifications were responsible for increasing the mitogenesis properties of breast cells, resulting in an amplification of the malignant process. This evidence was validated in ductal carcinoma in situ in 2019 by He et al. [100]. In 2012, Cox et al. [101] described in vitro that breast cancer cells were able to adapt to the bone microenvironment by developing osteomimetic characteristics. To further evidence the capability of breast cells to osteomimicry, Scimeca et al. [102] demonstrated, in 2019, that the presence of microcalcifications combined with the presence of activated monocytes induce an epithelial to mesenchymal transition. Furthermore, they proved an osteoblast phenotype acquisition of the cells producing hydroxyapatite by performing immunohistochemical analysis. This capacity of breast cells to develop osteomimetic properties were also reported in 2019 by O’Grady et al. [103]. In fact, they demonstrated the ability of breast cancer cell lines to mineralize, and doing so, proved the capacity of breast cancer cells to produce de novo microcalcifications in osteogenic conditions [103]. Furthermore, microcalcifications are described as an indirect sign of pathological process, such as inflammation, and, as described previously, inflammation is also associated with breast involution in both post-lactation and peri-menopausal contexts, but also in tumorigenesis and tumoral growth. These results taken together suggest that a lack of *SFRP1* in breast tissue could be responsible for osteoblastic differentiation, resulting in accumulation of new microcalcifications in the breast tissue.

## 6. Conclusions

We propose that the post-menopausal lobular involution initiation is a risk factor of breast cancer development due to the concomitant falling of estrogens and *SFRP1*. Lack of *SFRP1* induces a decrease of the adipogenesis needed for the replacement of epithelial cells in breast during lobular involution. Associated with that, the decrease of pre-adipocytes and adipocytes stem cells maturation, also due to the lack of *SFRP1*, increases dramatically the expression of adipokines resulting in an important inflammatory reaction. Furthermore, the low expression of *SFRP1* is responsible for an exacerbation of TGF-β sensitivity which is responsible for the chronic inflammation installation and the upregulation of Wnt molecule production. Increasing Wnt molecule quantity, added to the absence of *SFRP1* as a down-regulator of the Wnt signaling pathway, results in an in-chain reaction in which proliferation, adhesion, and migration are dramatically exacerbated. Furthermore, microcalcifications added to the Wnt signaling pathway over-stimulation result in osteoblastic differentiation of the cells and the production of new hydroxyapatite crystals in breast tissue (Figure 2B). Finally, it is essential to understand the exact role of *SFRP1* in both physiological lobular involution and tumor development. The *SFRP1* gene could become an important key player to optimize the protective effect of breast involution. Moreover, understanding the underlying mechanisms used by *SFRP1* to regulate mammary gland evolution could be crucial to prevent early breast tumorigenesis.

## Figures and Tables

**Table 1:** Dysregulation of *SFRP1* in human breast lesions or cell lines reported in the literature.

Alterations in *SFRP1*	Role of *SFRP1*	Population/Breast Cancer Subtypes	Signaling Pathway Involved in Tumorigenesis	Reference
Higher methylation and lower expression of *SFRP1* mRNA in tumors compared to non-tumoral tissues	Tumor-suppressor	NA */all subtypes	NA	Veeck et al. (2006) [28]
Under-expression of *SFRP1* mRNA and protein in tumors compared to non-tumoral tissues	Tumor-suppressor	NA/ductal and lobular carcinomas	NA	Dahl et al. (2007) [15]
Higher methylation, deletion, and under-expression of *SFRP1* mRNA in tumors compared to non-tumoral tissues	Tumor-suppressor	Indians/67% ER **/PR ***	Canonical Wnt signaling pathway	Mukherjee et al. (2012) [27]
Higher expression in TNBC ****compared to other BC subtypes	Higher sensitivity to chemotherapy in TNBC overexpressing *SFRP1*	NA/all subtypes	Wnt and TGF-β ***** signaling pathways	Bernemann et al. (2014) [32]
Under-expression of *SFRP1* mRNA in tumors compared to non-tumoral tissues	Tumor-suppressor	NA/basal-like	Canonical Wnt signaling pathway	Huth et al. (2014) [29]
Under-expression of *SFRP1* mRNA in tumors compared to non-tumoral tissues	Tumor-suppressor	NA/luminal-like HER2 positive	Non-canonical Wnt signaling pathway	
Estrogen-mediated signaling is differentially affected by the expression levels of Sfrp1 in mammary epithelial cells: Estrogen signaling and *SFRP1* expression	Tumor-suppressor	NA/luminal-like	Estrogen-mediated and Wnt signaling pathways	Gregory and Schneider (2015) [25]
Higher methylation and lower expression of *SFRP1* mRNA in tumors compared to non-tumoral tissues	Tumor-suppressor	Chineses /all subtypes	NA	Li et al. (2016) [26]
Copy number alterations induce under-expression of *SFRP1* mRNA in tumors compared to non-tumoral tissues	Tumor-suppressor	NA/all subtypes	NA	Zhu et al. (2018) [36]
Under-expression of *SFRP1* is associated with aggressiveness	Tumor-suppressor	NA/breast cancer risk continuum	NA	Kothari et al. (2018) [37]
Higher expression in begnin tissues compared to hyperplasias	Tumor-suppressor	NA/ductal and lobular hyperplasias	Estrogen-mediated and Wnt signaling pathways	Gregory et al. (2019) [30]

* NA = non-applicable, ** ER = estrogen receptor, *** PR = progesterone receptor, **** TNBC = triple-negative breast cancer, ***** TGF- β = transforming growth factor beta
